# Differential Methylation of Genomic Regions Associated with Heteroblasty Detected by M&M Algorithm in the Nonmodel Species* Eucalyptus globulus* Labill.

**DOI:** 10.1155/2016/4395153

**Published:** 2016-03-30

**Authors:** Rodrigo Hasbún, Carolina Iturra, Soraya Bravo, Boris Rebolledo-Jaramillo, Luis Valledor

**Affiliations:** ^1^Departamento de Silvicultura, Facultad de Ciencias Forestales, Universidad de Concepción, 4070386 Concepción, Chile; ^2^Centro de Biotecnología Vegetal, Facultad de Ciencias Biológicas, Universidad Andrés Bello, 8370146 Santiago, Chile; ^3^Department of Biochemistry and Molecular Biology, Pennsylvania State University, University Park, PA 16802, USA; ^4^Departamento de Biología de Organismos y Sistemas, Universidad de Oviedo, 33006 Oviedo, Spain

## Abstract

Epigenetic regulation plays important biological roles in plants, including timing of flowering and endosperm development. Little is known about the mechanisms controlling heterochrony (the change in the timing or rate of developmental events during ontogeny) in* Eucalyptus globulus*. DNA methylation has been proposed as a potential heterochrony regulatory mechanism in model species, but its role during the vegetative phase in* E. globulus* has not been explored. In order to investigate the molecular mechanisms governing heterochrony in* E. globulus*, we have developed a workflow aimed at generating high-resolution hypermethylome and hypomethylome maps that have been tested in two stages of vegetative growth phase: juvenile (6-month leaves) and adult (30-month leaves). We used the M&M algorithm, a computational approach that integrates MeDIP-seq and MRE-seq data, to identify differentially methylated regions (DMRs). Thousands of DMRs between juvenile and adult leaves of* E. globulus* were found. Although further investigations are required to define the loci associated with heterochrony/heteroblasty that are regulated by DNA methylation, these results suggest that locus-specific methylation could be major regulators of vegetative phase change. This information can support future conservation programs, for example, selecting the best methylomes for a determinate environment in a restoration project.

## 1. Introduction

Tree species are usually able to tolerate a wide range of environmental conditions. In fact, they are able to tolerate a wide range of environmental conditions and, in many cases, extreme seasonal changes [1]. Some of these organisms are able to manifest different phenotypes depending on the environment in which they grow. This phenomenon, called phenotypic plasticity, has been defined as a change in the phenotype expressed by a single genotype in different environments [2, 3]. Phenotypic plasticity evolves to maximize fitness when the environment is variable and increases with latitude [4–6].

Leaf heteroblasty is a significant and abrupt change in form and function that occurs over the maturation process (phase change from seedlings to reproductive individuals) of certain plants [7]. Characteristics commonly affected include leaf form, size, and arrangement. The earlier and later stages of leaf development are named juvenile and adult, respectively. In contrast to phenotypic plasticity, heteroblastic development does not depend on environmental cues. However, the timing or rate of heteroblastic changes, which can be referred to as a type of heterochrony—change of relative timing of events throughout development—can be modified by the environment [8]. Heterochrony has been implicated in plant evolution, because it can impact the physiology, phytochemistry, or resistance to pests and disease of certain plants [9, 10]; however it is a plastic response largely underexplored.

Phenotypic plasticity and heterochrony may interact to produce a pattern of variation in the leaf phenotype, even in organisms with little or no genetic diversity. Most endangered species have lower genetic diversity than related nonendangered species [11], and phenotypic plasticity and heterochrony together can increase the possibilities of adaption/persistence. Therefore, knowledge of the molecular mechanisms regulating both processes could open new alternatives to assist conservation programs.

Epigenetic regulation, in particular DNA methylation, plays an important biological role in plants, including timing of flowering and endosperm development [12]. Transgenerational inheritance of DNA methylation can mediate phenotypic plasticity via novel epialleles and phenotypes within populations/species [13]. In a review by Pascual et al. [3] it was shown that the coordination of genetic and epigenetic mechanisms mediated phenotypic variation in different plants. For example, in populations of* Arabidopsis thaliana* with experimental alteration of DNA methylation, the overall patterns of variability among the genotypes indicated that epigenetic changes could affect not only the short-term environmental responses of plants, but also the evolutionary potential of important traits and their plasticity [14]. Similarly, a recent study in invertebrates proposed that the absence of germline DNA methylation in genes involved in the response to fluctuating conditions facilitates phenotypic variation, which could contribute to increased adaptive potential [15]. In conifers it has been reported that environment influences a differential DNA methylation during embryogenesis, inducing differential priming of the embryos that causes differential capabilities to adapt to environment [16].

There is little evidence linking epigenetic regulation and heterochrony. Only a few studies focusing on epigenetic changes during leaf differentiation and development have been developed in* Arabidopsis* [17–19], rice [20], or pine [21] but none of these species are strongly heteroblastic. Environmental cues are perceived as input signals for the microRNA156/SQUAMOSA promoter-binding protein-like (SPL or SBP box) module and act as a quantitative developmental clock of phase transitions in plants [22, 23]. The evidence shows that sugars promote vegetative phase change through their effect on miR156 [24], but other endogenous factors could play additional important roles. The same authors proposed that heritable epigenetic modification of the miR156 precursor and/or additional chromatin structure alterations could regulate heteroblasty [24].

*Eucalyptus globulus*, a tree of Australian origin but cultivated worldwide, is strongly heteroblastic with clear differences between its juvenile and adult leaves [25]. Jordan et al. [26] found that genetic association of the timing of vegetative phase change with growth rate ranged from positive to negative at different sites. Early phase change may be favored in warm, wet environments to reduce damage produced by leaf fungi, but it may also be favored on exposed dry sites to increase form or plant structure by which it is protected from desiccation [27, 28]. Genome-wide DNA methylation maps of many model organisms have been reported, but in nonmodel organisms like* Eucalyptus* spp. the methylation patterns remain poorly studied. These types of maps can be applied to a wide range of biological problems, using the analysis of methylation differences between ecotypes or individuals within species [29–31].

The aim of this study is to set up the required methodology and assess the epigenetic changes (hypermethylation and hypomethylation) related to heteroblasty in* E. globulus*. Specifically, we want to detect differentially methylated regions (DMRs) between juvenile and adult leaves of* E. globulus*. DMRs are stretches of genomic DNA that have different DNA methylation patterns. Their natural variation could guide the conservation management of the species or the selection of individuals with potentially adequate methylomes—set of modifications of nucleic acid methylation in the genome of an organism—for a discrete environmental condition. We expect that the results and experience from this work could be used for the discovery of key regulators of heterochrony in future studies, which could be used to assist conservation programs of threatened species.

## 2. Materials and Methods

To investigate the molecular mechanisms governing heterochrony in* Eucalyptus globulus,* we applied the M&M algorithm [32] to identify DMRs related to heteroblasty of vegetative growth. We used the previously sequenced X46 clone (Mininco SA JGI Project ID: 401875). Ten ramets of the X46 clone were produced by cuttings, and a genetic trial was established in the commune of Renaico, province of Malleco, region of La Araucanía, Chile (latitude −37.67, longitude −72.59). Juvenile leaf material from five plants was harvested after 6 months at nodes 8 to 10, mixed, and stored at −70°C. Plants were grown until the vegetative phase change was evident (after more than 2 years of growth, average 45 nodes), and adult leaf material was collected. Genomic DNA was extracted using the DNeasy Plant Mini Kit (QIAGEN Inc.).

The methylation profiles were determined by DNA sequencing of enriched genomic libraries: (i) hypermethylome (the methylated part of the genome) using immunoprecipitation of methylated DNA (MeDIP-seq) and (ii) hypomethylome (the nonmethylated part of the genome) based on restriction enzymes sensitive to methylation (MRE-seq).

### 2.1. MeDIP-seq and MRE-seq Library Generation and Sequencing

MeDIP-seq is a large-scale purification technique used to enrich libraries for methylated DNA sequences. It consists of isolating methylated DNA fragments via an antibody raised against 5-methylcytosine (5mC). MRE-seq utilizes a combination of methyl sensitive enzymes to enrich libraries for unmethylated DNA sequences.

Libraries were generated as previously described in Li et al. [32], with minor modifications. For MeDIP-seq, 3500 ng of isolated DNA was sonicated using 26 pulses of 10 s ON/20 s OFF (Sonic Dismembrator model 100, Fisher Scientific) to a fragment size of 100–500 bp, end processed, and ligated to paired-end adapters using NEXTflex PCR free DNA Sequencing Kit (Bioo Scientific). After size selection of 166–566 bp using Agencourt AMPure XP (Beckman Coulter), DNA was heat denatured and then immunoprecipitated using Methylated DNA IP Kit (Zymo Research), using a mouse monoclonal anti-5-methylcytosine antibody according to manufacturer's instructions. DNA was then purified with Agencourt AMPure XP (Beckman Coulter) and eluted in 25 mL resuspension buffer (10 mM Tris-HCl, pH 8.5). DNA was amplified by 12 cycles of PCR with the standard Illumina index primers. For MRE-seq, three digestion reactions (HpaII, Acil, and Hin6I; Fermentas) were performed in parallel, each with 600 ng of DNA. Ten units of enzyme (except Acil, which uses five units) were initially incubated with DNA for 3 h, and then additional five units of enzyme were added to the digestion for a total of 6 h of digestion time. Digested DNA from the different reactions was combined and purified using ChiP DNA Clean & Concentrator*™* (Zymo Research). The purified DNA was end processed and ligated to single-end adapters using NEXTflex PCR free DNA Sequencing Kit (Bioo Scientific). After size selection (166–566 bp) with Agencourt AMPure XP (Beckman Coulter), the DNA was amplified by PCR for six cycles.

MeDIP and MRE libraries were sent to the DNA Sequencing Facility of the Biotechnology Center at the University of Wisconsin. Samples were sequenced on an Illumina HiSeq machine, yielding a total of 204 million MeDIP-seq reads and 236 million MRE-seq reads. The reads were mapped to the latest* Eucalyptus grandis* genome assembly (v2.0) [33], using BWA-MEM Li [34] with default settings.

### 2.2. Use of M&M Algorithm to Detect DMRs

We used an algorithm named M&M [32] that integrates data from both MeDIP-seq and MRE-seq to detect DMRs. M&M is available as an R package called “methylMnM.” Briefly, M&M integrates MeDIP-seq and MRE-seq by dynamically scaling, normalizing, and combining the datasets and provides exact *p* value and *q*-value for different sample comparison. The coverage of MeDIP and MRE sequencing data and genomic CpG information were calculated for each 2000 bp genomic bin. Before applying the M&M method, we generated two input files: (1) CpG sites of each window and (2) MRE-CpG sites of each window. To generate file (1) we used the script fasta2bed.py (Computational Genomics Analysis Tools CGAT 0.2.3), and to calculate (2) we used a script created specifically for this project (discussed at https://www.biostars.org/p/86480/). DMRs between developmental stages were identified using the M&M algorithm with default parameters in the R environment (version v.2.12.1). Briefly, the coverage of MeDIP and MRE sequencing data and genomic CpG information were calculated for each 2000 bp genomic bin. Scaffolds were excluded from the analysis. DMRs with a *q*-value of 1E − 7 were selected for analysis. Several statistical functions are implemented in the methylMnM package: “MnM.test()” to calculate the probability that the methylation levels of the two samples within each bin were different, “MnM.*q*value()” to estimate *q*-values based on all the *p* values, and “MnM.select-DMR()” to select significant DMRs based on a cutoff of *q*-value < 1E − 4. The output files contained genomic locations of statistically significant DMRs and their MeDIP-seq and MRE-seq values (in RPKM), as well as *p* values and *q*-values. The absolute values of genomic regions are negative log_10_-transformed *q*-values. If the value is negative, it represents hypermethylation in the vegetative juvenile sample and hypomethylation in the vegetative adult; if the value is positive, it represents hypomethylation in the vegetative juvenile sample and hypermethylation in the vegetative adult. Mapping results and detected DMRs were visually inspected with the GenomeView software [35] using default parameters.

## 3. Results and Discussion

### 3.1. Sequencing Results

For each developmental stage, we constructed one sequencing library using two complementary technologies: MeDIP-seq and MRE-seq. These libraries were sequenced to generate 446 million reads in total (Table 1), of which 387 million were mapped to the* E. grandis* genome covering at least 50%. We note that MeDIP-seq data has less mapping efficiency. Li et al. [32] explained this because relatively more MeDIP-seq reads are derived from repetitive regions of the genome, which are often heavily methylated. Some reads from repetitive regions cannot be mapped uniquely. In line with expectations MRE-seq scores were inversely correlated with MeDIP-seq scores.

**Figure 1:**
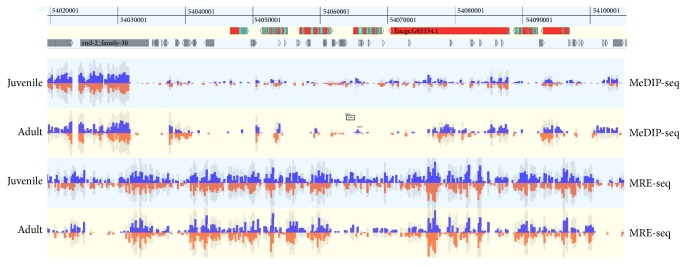
Epigenome view (GenomeView software) of a 138.7 kb region of chromosome 07 (54,020,001–54,100,001) of* Eucalyptus grandis* reference genome. MeDIP-seq libraries and MRE-seq libraries covered largely nonoverlapping and short overlapping regions.

The two sequencing signals covered nonoverlapping regions as expected, but some adjacent genomics regions showed overlapping signals (Figure 1).

### 3.2. Identification of DMRs

The main aim of this work was the determination of the local DNA methylation changes between two developmental phases (i.e., vegetative juvenile and vegetative adult). We only considered CpG methylation and applied the M&M algorithm to our data to identify DMRs. This allowed us to find a total of 1090 putative DMRs (*q*-value < 1E − 4) between the two developmental stages. More than 70% of DMRs showed increasing DNA methylation levels from vegetative growth phases juvenile to adult. These preliminary results suggest that locus-specific methylation patterns could be an important feature of vegetative heteroblasty control. This increment in the number of methylated regions agrees with the reduction in the number of genes and proteins that is observed in mature leaves (when they reach their full physiological competence) compared to leaves in proliferative stage [36, 37] which have been also related to an increase of global DNA methylation in conifers [21, 38] and* Arabidopsis* [39].

In future work, candidate DMRs related to genes involved in plastic responses will be validated using more genotypes of* E. globulus* with heterochronic responses in the expression of heteroblasty. Using quantitative techniques for quantifying methylation (e.g., bisulfite/sequencing of specific genomic locus) we hope to find DMRs that could explain or predict heterochronic responses. Knowledge of genomic loci that regulate heterochrony in* E. globulus* will improve our understanding of molecular mechanism of this process and can support future conservation programs, for example, selecting the best methylomes for a specific environment in a restoration project. This complete workflow could be easily applied to other nonmodel species with the only requirement of a reference genome. Given that the cost of DNA sequencing is falling [40] and bioinformatics facilities for assembly of massive sequences are increasing quickly [41], generating a reference genome for almost any species is becoming more affordable. The latter also is valid for threatened species.

The introduction of a workflow allowing the study of the epigenetic regulation at genome level will provide new insight that goes further than the mere description of the genes that are regulated during plant development or environmental adaption. For instance, having the possibility of tracking epigenetic changes in the form of metastable epialleles—alleles that are variably expressed in genetically identical individuals due to epigenetic modifications established during early development and are thought to be particularly vulnerable to environmental influences—will allow us to add a new source of variation, which is particularly important for threatened species, which usually exhibit a very low genetic diversity [42, 43]. Although these studies can be done routinely in model species [44, 45], it is particularly difficult to adapt wet and* in silico* methodologies for nonmodel like trees, since tissues are rich in polyphenols and other contaminant molecules requiring optimizations (e.g., [46–48]) and at the same time bioinformatics pipelines (gene prediction, characterization, and annotation algorithms) still require improvements [49]. In this study we describe a workflow aiming at achieving results comparable to those obtained in* Arabidopsis* in terms of quality of libraries and quantity of reads. This procedure will allow future studies employing a higher number of samples and experimental situations that permit addressing important issues related to conservation and management of threatened species such as restoration and translocation. Furthermore, the integration of this regulatory layer together with other omic levels has been proved to be useful for explaining adaptive divergences (see Meijón et al. [50] for an example of the power of high-throughput approaches to explore natural variation in tree species).

## 4. Conclusion

These preliminary results suggest that locus-specific methylation process could be an important feature of vegetative heteroblasty control in* E. globulus*. The workflow set up in this project opens a promising future for discovering DNA methylation patterns among different tissue types, cell types, and individuals that will help us to explain phenotypic plasticity and adaption capabilities through the basis of a divergent epigenetic regulation. High-throughput epigenomic technology and analytical tools used in this study could be applied to population-based studies of nonmodel plants but principal challenges are experimental design, data analysis, and interpretation of results. The implementation of epigenetic fingerprinting as a support tool in restoration and conservation projects of threatened species requires the discovery of loci involved in adaptive variation.

## Figures and Tables

**Table 1:** Summary of mapping statistic from MeDIP-seq and MRE-seq libraries of juvenile and adult leaves.

	Total fragments (Tf)	Mapped fragments	% of Tf	Uniquely mapped fragments	% of Tf	Nonredundant uniquely mapped fragments	Genome coverage (%)
MeDIP-seq-juvenile	1.37*E* + 8	1.17*E* + 8	85.4	6.74*E* + 7	49.1	6.79*E* + 6	52.3
MeDIP-seq-adult	0.70*E* + 8	0.57*E* + 8	82.0	2.48*E* + 7	35.4	6.72*E* + 6	38.3
MRE-seq-juvenile	1.14*E* + 8	1.02*E* + 8	89.5	7.91*E* + 7	69.4	9.6*E* + 6	57.0
MRE-seq-adult	1.25*E* + 8	1.11*E* + 8	88.8	8.83*E* + 7	70.6	6.34*E* + 6	49.2

Total	4.46*E* + 8	3.87*E* + 8	86.7	2.60*E* + 8	58.3	2.95*E* + 7	
